# Sanitary Administration in Ireland

**Published:** 1907-07-13

**Authors:** 


### Public Health and Hygiene.

### SANITARY ADMINISTRATION IN IRELAND.

Sir Charles A. Cameron, Professor of Hygiene and Chemistry, R.C.S.I., and Chief Medical Officer of Health for Dublin, read a paper at the Conference of the Royal Sanitary Institute held in Dublin on June 25, in which he thus sums up sanitary administration as practised in the Sister Isle:—

"The Sanitary Acts are administered in Ireland by the following bodies. First, the Local Government Board, the supreme public health authority of the country; second, the county boroughs; third, the urban district councils; fourth, the rural district councils. The Local Government Board has certain powers vested in it by statute, which enable it to supervise the boards of guardians who have charge of the pauper poor, sick or well. The Board must approve of the appointment and dismissal of officers of the guardians; its auditor examines their accounts and makes surcharges if payments of money have been illegally made. It must approve of the appointment and dismissal of sanitary officers, and of their salaries and increases of salaries. This practically secures fixity of tenure to the medical officers of health, who are appointed by the sanitary authorities, in which respect they are in a more secure position than the medical officers of health in England and Wales.

"The borough councils are empowered to appoint medical superintendent officers of health, executive sanitary officers, and sanitary sub-officers (another name for inspector of nuisances or sanitary inspector). They can establish hospitals and work them, or contribute to their maintenance, or do both.

"The Irish Public Health Act of 1875 constituted all the Poor-law medical officers *ex-officio* medical officers of health. They were paid by the boards of guardians; but when their districts were situated in towns having sanitary authorities, their salaries were fixed, though not paid, by those authorities. This anomaly ceased on the passage of the Irish Local Government Act, which transferred the payment of the salaries of the medical officers of health from the boards of guardians to the governing bodies of the counties and boroughs, and of the newly created rural district councils.

"It is now generally conceded that it was a mistake to have converted *nolens volens* the dispensary physicians into medical officers of health. So far as the large towns are concerned, the district medical officers of health perform, on the whole, very good sanitary work. In the rural districts they are handicapped very largely. They have not efficient sanitary sub-officers. The rural district councillors are practically the boards of guardians, who elect and pay them as dispensary physicians or medical officers of the workhouses. The health officers, whenever they make sanitary reports, are not unlikely to give offence to some one or other of the rural district councillors. This is particularly the case as regards the hygiene of the dairy and farmyard. A considerable proportion of the milk supplied to the towns comes from the country. The sanitary sub-officer, who is generally also the relieving officer, and who has a salary of only a few pounds a year, can hardly be expected to give much attention to the hygiene of the dairy and cowsheds. He is not qualified by the possession of a certificate of competency to act as a health officer granted by such bodies as the Royal Sanitary Institute. I think it may safely be assumed that in the greater number of the rural districts in Ireland the sanitary laws are practically a dead letter.

"Whether or not the *ex-officio* medical officers of health should cease to exist, the county council ought to be empowered to appoint medical officers of health and sanitary sub-officers. If the officers' functions ceased, the sanitary staff of the county would have to be larger than if there were no district medical officers. In England and Scotland, as well as in Ireland, there are district medical officers of health; but that did not prevent the establishment of county officers, with powers to act in every district and to review the proceedings of the local authorities."

Sir Charles Cameron then suggested that the Conference of the Sanitary Institute should urge the appointment of county medical officers and a staff of fully qualified sanitary inspectors, and a motion to this effect was carried *nem. con.* Having regard to the peculiar character of the public health service, this would certainly seem to be the best solution of the problem of sanitary administration in Ireland. Were this done, there would then be no need to interfere with the existing *ex-officio* medical officers of health; but as vacancies occurred it might be enacted that their duties should, where it was desired, devolve upon the county medical officers.

